# Sutureless Thyroidectomy With Intraoperative Neuromonitoring and Energy-Based Device Without Sternotomy for Symptomatic Substernal Goiter Harboring Thyroiditis of Gland Parenchyma

**DOI:** 10.7759/cureus.16258

**Published:** 2021-07-08

**Authors:** Demet Sengul, Ilker Sengul, Tuncer Ozturk

**Affiliations:** 1 Pathology, Giresun University Faculty of Medicine, Giresun, TUR; 2 Endocrine Surgery/General Surgery, Giresun University Faculty of Medicine, Giresun, TUR; 3 General Surgery, Giresun University Faculty of Medicine, Giresun, TUR

**Keywords:** thyroid, substernal goiter, sutureless thyroidectomy, ligament of berry, recurrent laryngeal nerve, intraoperative neural monitoring, ionm, thyroidology, suspicious for malignancy, differentiated thyroid carcinoma

## Abstract

Since substernal goiter first being described by Haller in 1749, a consensus on the definition of this entity has not been ensured, yet. Despite substernal goiter or retrosternal goiter is delineated as an enlarged thyroid gland with a component extending into the mediastinum, at least 10 definitions have described being able to depict the most accurate definition for substernal goiter. Of note, no consensus still has been declared on the therapeutic management of asymptomatic substernal goiter. It should be pointed out that, the American Association of Endocrine Surgeons (AAES), Guidelines for the Definitive Surgical Management of Thyroid Disease in Adults reported approximately 9% to 13% of substernal goiters are being harbored thyroid malignancy. The following vignette case describes the clinical features of a symptomatic substernal goiter with chronic lymphocytic thyroiditis, her treatment by sutureless total thyroidectomy with intermittent-intraoperative neural monitoring (I-IONM), and the energy-based device without sternotomy, and the response of an adult who presented with a family history of malignant histopathology, differentiated thyroid carcinoma, which was exposed to the postoperative radioactive iodine ablation. We may recommend dividing the branches of superior thyroid arteries and the superior thyroid veins individually and exploring the fibrous Ligament of Berry, the true Ligament of Berry, with its safe interrelation to the inferior laryngeal nerve, finically, which may be regarded as different peas in a pod in a complete sutureless thyroidectomy for substernal goiter with thyroiditis in thyroidology.

## Introduction

The term goiter refers to enlargement of the thyroid gland which is concealed with the thin muscles, subcutaneous fatty tissue, and delicate skin of the neck, those notwithstanding sufficiently while growing. As the gland, one or both the lobes descends through the thoracic inlet into the thoracic cavity, then it is termed as substernal or retrosternal, subclavicular, and mediastinal goiter [1]. On the other hand, Hsu’s definition, clinical and radiologic inferiority of the thyroid regarding the manubrium sterni; Kocher’s definition, perpetual retrosternality of some portions of the thyroid; Torre’s definition, the lower border of the gland is being permanently remaining below the sternal notch; Eschapase’s definition, location of the gland in the mediastinum, entirely or partially provided that the inferior border of the gland is three cm inferior, at least, in hyperextension of the neck; Lahey’s definition, thyroidectomy, necessitating to entrance into the superior mediastinum; Lindskog definition, expansion of the thyroid to the 4th thoracic vertebra; Crile’s definition, a gland reaching the arcus aorta; Katlic’s definition, greater than 50% of the gland residing substernal, have been described to date [2]. The etiology of the substernal goiter is identical to the cervical-located goiter. De novo substernal goiter, in the other words ectopic thyroid tissue in the chest, is seldom that accounts for merely 2% of all substernal goiter [3]. The deficiency of epidemiological studies inherently has led to an unknown prevalence of substernal goiter in the general population. However, it is four times more common in females and more frequently diagnosed after age 50 years [1,4].

Herein, it is purposed to present our case, underwent a total thyroidectomy with intermittent-intraoperative neural monitoring (I-IONM) and energy-based device without sternotomy for symptomatic substernal goiter harboring thyroiditis with a family history of radioactive iodine (RAI) ablated differentiated thyroid carcinoma, papillary thyroid carcinoma.

## Case presentation

A 57-year-old female admitted with a history of difficulty in swallowing, shortness of breath, and hoarseness. Her personal medical history has included diabetes mellitus, hypertension, hyperlipidemia, atrial fibrillation, and familial mediterranean fever while her family history, her sister, who was comprising differentiated thyroid carcinoma, papillary thyroid carcinoma, underwent total thyroidectomy with a subsequent RAI ablation. Her vital signs were recorded within normal limits and she had no history of radiation exposure in the first two decades of her life. She was in a euthyroid hormonal status with chronic lymphocytic thyroiditis, compatible with the relevant levels of thyroid antibodies. A physical examination at the time of admission revealed a multinodular goiter with a prominent palpable firm nodule of 25 x 20 mm at the left lobe inferior.

Diagnostic imaging

**Figure 1:**
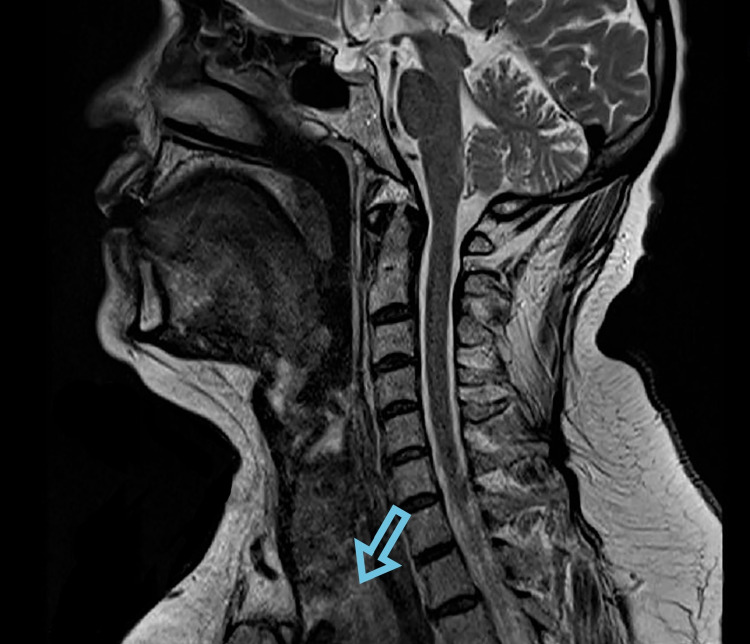
The expansion down of the left inferior lobe of thyroid (arrow), the sagittal section, diagnostic imaging of the neck MRI. MRI: magnetic resonance imaging.

**Figure 2:**
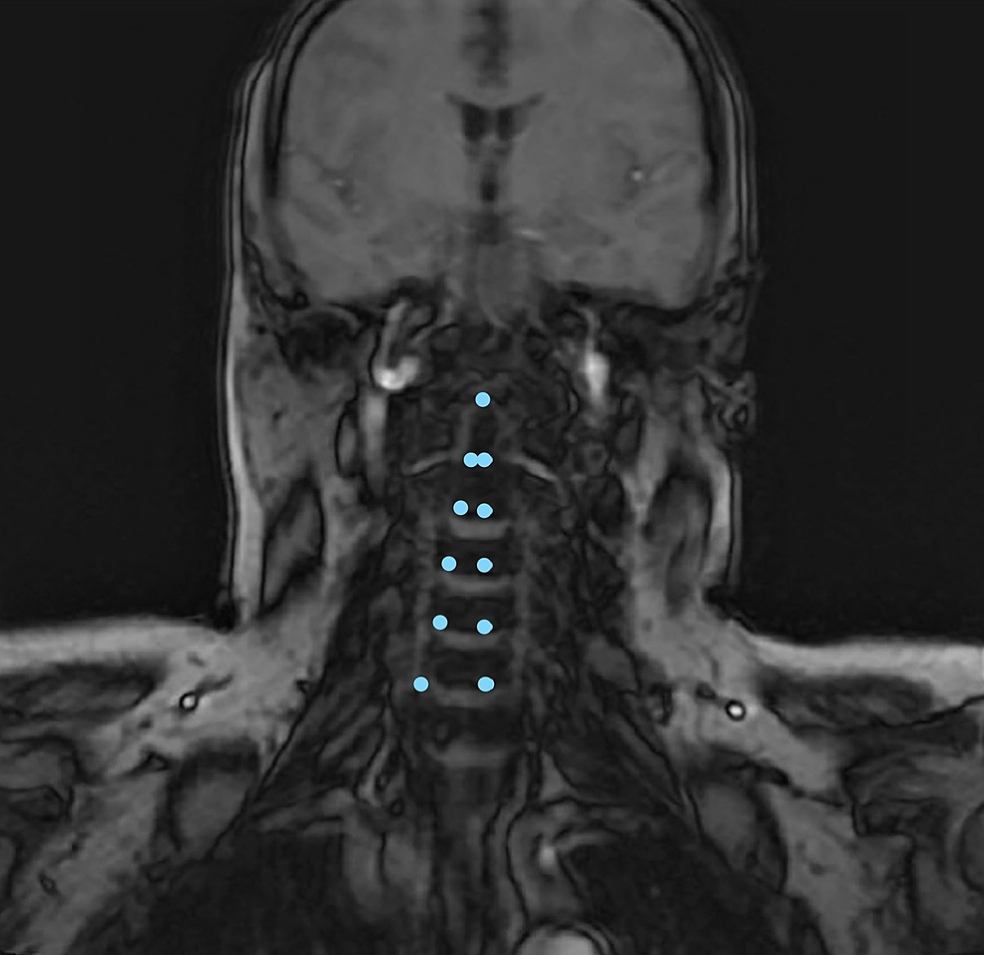
The displacement of the trachea to the right lateral (angular pointed lines), the coronal section, diagnostic imaging of the neck MRI. MRI: magnetic resonance imaging.

Her neck B-Mode sonography exposed the multiple nodules, the larger of which in the left lobe inferior, hyperechoic solid nodule of 26 x 20 mm in diameter with well-demarcated hypoechoic halo, without able to detect its most proximal border. The neck magnetic resonance imaging (MRI) revealed a multinodular goiter with the largest nodule, 24 x 20 mm in diameter, in the left lobe inferior, expanding retrosternally while her thorax MRI put forward the left lobe was descending to the left brachiocephalic vein and the trachea was displaced to the right. Her video laryngoscopy revealed no pathologic finding (Figures 1, 2).

Surgical procedures

A total thyroidectomy by the collar incision without a median sternotomy with I-IONM and the energy-based device was decided in order of facing with symptomatic substernal goiter, including the bilateral multiple nodules, that did not extend beyond the left innominate vein proximally, such the vital organs like as the arcus aorta. The informed consent was received for both her treatment and the present article, preoperatively. To this end, it was practiced on the ‘Iowa Head and Neck Protocols for Thyroidectomy and Thyroid, Iowa, the US' [5] for most of the surgical procedures. For these purposes, she was brought to the operating room and placed on the operating table in the supine position. She was then transorally intubated and a skin crease incision was designed and drawn at one fingerbreadth above her clavicles. Thereafter, a shoulder roll was placed behind the scapulae and a mini roll behind the posterior aspect of the neck, then her hyperextended neck was prepared with chlorhexidine and draped in a sterile fashion. A 15-blade scalpel was used to perform the planned and drawn beforehand Kocher's transverse collar incision, which was carried down through the platysma to the level of the strap muscles of the neck, after administrating the agents of lidocaine 1% and epinephrine 1:100,000 into the incision line. The subplatysmal flaps were subsequently elevated through accessing its avascular space and underlying areolar tissue plan. Paying attention to the preservation of the anterior jugular veins, the strap muscles were split in the midline raphae, the linea alba cervicalis, the fascia of sternothyroid muscle was divided, and the capsular blood vessels of the gland were pointed out. After constituting a proper cleavage plan, the sternothyroid and sternothyroid muscles were retracted laterally to the gland, leading to the thyroid were explored without being supposed to dissection of the strap muscles. Firstly, the left middle vein was explored and divided to be able to provide safe and bloodless maneuvers of the left lobe of the gland. In dissecting around the left superior pole of the thyroid, the branches of the left superior thyroid artery and left superior thyroid vein were dissected separately in order not to harm the left superior laryngeal nerve, and the left superior parathyroid gland was revealed and was dissected away from the lobe. The thyroid gland was then retracted anteriorly and medially in order of facilitating the exposure of the left tracheoesophageal groove. The left inferior thyroid artery was exposed and the left recurrent laryngeal nerve (RLN) was identified and preserved by using its present anatomic association, i.e. crossing it posteriorly. The mentioned situation was also confirmed by I-IONM. The gland was then retracted superiorly and laterally in order of releasing the left inferior pole. Charles Proye’s Toboggan Technique was used to facilitate the dissection and retraction out of the left inferior lobe from the surgical bed by slackening its remaining attachments and division of the left inferior thyroid veins. Herewith, the left inferior parathyroid gland was exposed and preserved. The thyroid was carefully examined after the removal process with LigaSure Small Jaw (LSJ) (LF1212A) in order of being convinced for not harboring any evidence of parathyroid tissue on it. Afterwards, the right side was dissected and spared in a similar fashion with the relevant exposures of the branches of right superior thyroid artery and right superior thyroid vein, right superior laryngeal nerve, right superior and inferior parathyroid glands, right inferior thyroid artery and vein, and right recurrent laryngeal nerve (RLN) by using LSJ (LF1212A) and I-IONM. Both the RLNs, per se, were not lying over the enlarged gland as being possible in case of a substernal goiter. What follows both thyroid lobes with the isthmus and pyramidal lobe were resected with the aid of the I-IONM and energy-based device, the mini roll behind the posterior aspect of the neck was removed in order to attenuate the collar tension, then the wound was profusely and meticulously irrigated with the sterile saline and the relevant haemostasis was provided. The mentioned field of the surgical procedure had preserved till the anesthesiologist recognized the positions of the vocal cords. Meanwhile, the entire gland was examined carefully, particularly whether harboring any parathyroid gland/tissue or not. While no evidence of any parathyroid gland/tissue and lymphadenopathy in the level VI, cervical compartment, was notified, a 15-French Jackson-Pratt drain was inserted and secured to the skin with 3-0 non-absorbable suture. The wound was closed by reapproximating the strap muscles and platysma with deep 3-0 vicryl stitches while the skin by a running 5-0 monocryl subcuticular continuous stitch with the benzoin and steri-strips, placing over the collar incision. Eventually, she had tolerated the procedure well and extubated in the operating room uneventfully. Herein, she transferred to the post-anesthesia care unit, subsequently. No intra-, peri-, and postoperative complications and/or morbidity have developed. Ultimately, she demonstrated improved symptoms and discharged home on the hospital day one.

Histopathologic evaluations

Macroscopically, the three colloid-rich nodules, 24 x 20 x 20 mm in diameters, at the right lobe and one colloid-rich nodule, 38x26x24 mm in diameter at the left lobe of the gland were exhibited. Microscopically, the nodular architecture, entirely enveloped by thin fibrous capsule including the pale staining cuboidal cells with round inconspicuous nucleoli and in micro- and macrofollicular patterns without mitose and the extensive lymphocytic infiltrate with germinal center formation, atrophic follicles together with Hürthle cells, fibrosis, not extending beyond the capsule were revealed. Immunohistochemically, Hector Battifora Mesothelial-1 (HBME-1), Cytokeratin-19 (CK-19), and Galectin-3 negativity emerged. Herein, the ultimate histopathological diagnoses of the case revealed Hashimoto’s thyroiditis, adenomatous hyperplasia, and two reactive lymph nodes at the central compartment, VIth cervical compartment.

## Discussion

Substernal goiter is simply defined as an enlargement of the thyroid gland with a component extending to the mediastinum since first being described anatomically by Haller in 1749. However, the first written account for the successful removal was that of Klein in 1820. As such, Hedenus declared a case series of removals without the death of six substernal goiters, leading to suffocation, in 1821 [6].

Primary substernal goiter is a congenital and rare phenomenon, accounting for less than 1% of all substernal goiters. It is derived from an anterior mediastinal ectopic thyroid tissue while 10%-15% may have possession of mid- or posterior mediastinal location. Secondary substernal goiter, sliding down cervical goiter, is the most common type of substernal goiter and a kind of endothoracic/mediastinal extension of cervical goiter. The arterial supply of primary substernal goiter comes usually from the mediastinal vessels while secondary one from the inferior or superior thyroid arteries [2,7]. In addition, approximately 10%-20% of substernal goiters, have undergone thyroidectomy, are derived from the remnants of thyroidectomy rather than the total, ipsilaterally or hemithyroidectomy, contralaterally [2,8]. Cough, short breathing, asphyxia, tracheomalacia, dysphagia, descendent esophagus varices, superior vena cava syndrome, Pemberton’s sign, venous thrombosis, cerebral ischemia, RLN paralysis, phrenic nerve paralysis, Horner syndrome, and chylothorax are involved in the compression symptoms of substernal goiter. Thyrotoxicosis with its significant symptoms emerges from autonomous transformation of the thyroid nodules over time. The mentioned hormonal status in substernal goiter is usually associated with the elderly and possession of the compression symptoms [2]. Differential diagnosis of substernal goiter comprises thymoma and thymic carcinoma, bronchogenic cyst, teratoma and germ cell tumors, lymphoma, pericardial cyst, ganglioneuroma, and other neurogenic tumors of the posterior mediastinum [1].

Of note, as talking over prognosis, symptomatic substernal goiter is undergone to surgery, hampering the study of natural history and so, its accurate evaluation. For this reason, prospective studies are convenient for surveillance in the mentioned aspects [1]. However, the substernal goiter may lead to some crucial complications such as obstruction of trachea/upper airway, compression of venous vasculature, cervical sympathetic chain, Horner syndrome, and other surrounding intrathoracic vital structures, phrenic nerve paralysis, cerebrovascular steal syndrome, jugular vein thrombosis, and superior vena cava syndrome [1]. Recently, the American Association of Endocrine Surgeons (AAES) Guidelines for the Definitive Surgical Management of Thyroid Disease in Adults reported approximately 9% to 13% of substernal goiters are being harbored malignancy. The mentioned current AAES Guidelines also remarked the cases of substernal goiter with a positive Pemberton’s sign, per se, warrant thyroidectomy [3].

In case of hormonal instability, intervening to provide a euthyroid hormonal status is antecedently essential. Surgery should be addressed for symptomatic substernal goiter, such as with compression symptoms, venous congestion, tracheal deviation or narrowing, and suspicion for malignancy while it is controversial for asymptomatic substernal goiter [1,2]. For this purpose, a close follow-up is a prerequisite in terms of any alteration in the size of the gland, tracheal diameter, and hormonal status in such cases. Besides, (i) growing down and compressing the intrathoracic structures for substernal goiter, eventually, if left untreated, (ii) harboring undiagnosed thyroid cancer focus/foci within the gland, (iii) possibility of more difficult surgical procedure(s) with a presumptive higher complication rate as the age progresses have been asserted as the proponents of surgical approach for asymptomatic substernal goiter [1].

The therapeutic approach can be evaluated in two subdivisions: (i) with malignancy, surgery is a unique recommendation and (ii) with benign conditions, (iia) thyroid hormone therapy, (iib) radioiodine, and (iic) surgery [9]. L-T4 therapy, suppressive therapy, is notified as ineffective, possessing a limited role in diffuse goiters in the young [1,9,10]. In the event of cases inappropriate for surgery, RAI ablation is a unique alternative therapy before the appearance of airway obstruction [11,12]. Hegedüs and Bonnema [9] reported that the radioiodine-related goiter shrinkage by 30-50% was proclaimed. However, Can and Nagalli [1] stated that radioiodine ablation can obscure/overlook thyroid carcinoma and radiation-induced thyroiditis in the closed thoracic cavity may theoretically lead to respiratory compromise.

Preoperative laryngoscopy must be performed, serum calcium should be inspected in order of excluding primary hyperparathyroidism [2] and antithyroid therapy should be administrated two weeks prior to surgery. Intercalarily, an inorganic iodine solution should be administrated 7-10 days before surgery in order to avoid a probable thyroid storm without essentiality for the cases with solely subclinical hyperthyroidism [1]. Despite the vast majority, more than 95% [2], around 98% [10], of substernal goiter was enunciated as could being safely removed transcervically, an entailment of a cardiothoracic back up should be evaluated preoperatively [13]. The current AAES Guidelines for the Definitive Surgical Management of Thyroid Disease in Adults recommend total thyroidectomy for bilateral goiter principally, however some clinical scenarios that total thyroidectomy may not be feasible or probable are remarked [3]. Extracervical approach may range from partial sternotomy to thoracotomy, has stated as requisite in 1% to 7.6%, frequently used in around 2% [9], of the surgical procedures for the indicated substernal goiters [1,14]. Mercante and colleagues [15] suggested a three-tiered classification system for substernal goiter: (i) Grade I, the inferior border of the thyroid is being above the aortic arch, (ii) Grade II, the inferior border of the thyroid is being between the convex and concave parts of the aortic arch, (iii) Grade III, the inferior border of the thyroid is below the concave part of the aortic arch. The demand for an extracervical approach was remarked significantly higher for Grade II and III, Mercante and colleagues [15], substernal goiters. Our vignette case with symptomatic substernal goiter was harboring the most inferior border the left lobe which was not extending beyond the left innominate vein, Grade I, Mercante and colleagues’ classification. A therapeutic approach in terms of surgery is recommended for symptomatic substernal goiter, i.e. possessing compression symptoms, venous congestion, tracheal deviation or narrowing, and suspicion for malignancy [16-20].

On account of the thyroid gland has been stated as a highly vascularized organ, accomplishing meticulous hemostasis is crucial in order to refrain from critical complications. The recent introduction of various surgical energy devices for the surgical procedures of the thyroid gland, such as LSJ vessel sealing system and harmonic devices, induce the providers to propound some advantages, like the ease of surgical procedure, less blood loss, shorter operation time, and shorter hospitalization. LSJ utilizes a combination of energy and pressure applications for the fusion procedure of the vessel and tissue bundles and seals them permanently up to seven mm in diameter without necessitating dissection and isolation. The seals can withstand up to three times the normal systolic pressure and the average seal cycle is two-four seconds. After the seal cycle is completed, the feedback-controlled response system automatically de-energizes without being supposed to the relevant assumption. Herewith, coagulation occurs as collagen and elastin are denatured to form a seal. Some authors asserted that utilization of most used energy-based devices; Ultrasonic Harmonic Focus Scalpel, LSJ, and Thunderbeat Open Fine Jaw have been alleged by some authors as effective and even safe in both hemostasis and dissection alongside the course of the inferior laryngeal nerve and even within the suspensory Ligament of Berry. In addition to hereinabove, application of intermittent or continuous intraoperative neural monitoring, I-IONM, C-IONM, respectively, for the mentioned surgical collar procedure hereinbefore has momentously been recommended [16].

Herewith, our vignette case with symptomatic substernal goiter harboring thyroiditis with a family history of radioiodine ablated differentiated thyroid carcinoma was administrated as a treatment modality of an LSJ-assisted sutureless total thyroidectomy with I-IONM.

## Conclusions

The vignette case with a symptomatic substernal multinodular goiter, living in an iodine deficiency area, had possession of thyroiditis with a family history of RAI ablated thyroid malignancy. As an aide-memoire in the management of symptomatic substernal goiter, harboring compression symptoms, venous congestion, tracheal deviation or narrowing, and suspicion for malignancy, emphasizing the entailment of surgical approach has had importance. The present case had a symptomatic substernal multinodular goiter with the most inferior border of its left lobe was not extending beyond the left innominate vein, Grade I, Mercante and colleagues’ classification. Herein, we opt for performing the total thyroidectomy with a transverse collar incision by using LSJ and I-IONM, considering to split the branches of superior thyroid arteries and the superior thyroid veins, separately. Of note, we dissected and divided the superficial vascular layer of the Ligament of Berry meticulously in order to expose the RLN lying on and also lateral to the underlying fibrous Ligament of Berry, also known as the true Ligament of Berry. Last but not least, in our opinion, dividing the branches of superior thyroid vasculature individually and exploring the true Ligament of Berry with its safe interrelation to the RLN punctiliously may be considered as peas in a pod in a complete sutureless thyroidectomy for substernal goiter.
